# Gone but not forgotten: dynamics of sperm storage and potential ejaculate digestion in the black soldier fly *Hermetia illucens*

**DOI:** 10.1098/rsos.241205

**Published:** 2024-10-30

**Authors:** Frédéric Manas, Harmony Piterois, Carole Labrousse, Laureen Beaugeard, Rustem Uzbekov, Christophe Bressac

**Affiliations:** ^1^ Insect Research Biology Institute (IRBI), UMR CNRS 7261 University of Tours, Tours 37200, France; ^2^ Plateforme IBiSA de Microscopie Electronique, University of Tours and CHRU of Tours, Tours 37200, France

**Keywords:** spermathecae, sperm viability, successive egg-laying, sperm plug, nuptial gift

## Abstract

Understanding the dynamics of sperm storage is essential to unravel the complexity of post-copulatory sexual selection processes in internally fertilized species. This physiological process goes from sperm transfer during copulation to its use for fertilization. In this context, the spatiotemporal dynamics of sperm storage were described in the black soldier fly (BSF) with fluorescence and transmission electron microscopy (TEM). BSF females have compartmentalized spermathecae with a transfer compartment, the fishnet canals, and a storage compartment, the reservoirs. Spermatozoa were counted both during and after mating in the two compartments. In addition to seminal fluids, the male transfers a mass of sperm in the fishnet canals, then only 49% of the transferred spermatozoa reach the reservoirs over two days. TEM observations of the fishnet canals revealed potential digestive functions, explaining the decline in the number and viability of spermatozoa in this compartment but not in the reservoirs. After one mating, females laid up to three fertile clutches, showing no constraints on sperm quantity or quality. Spermatic and ultrastructural investigations strongly suggest that BSF ejaculate acts both as a sperm plug and as a nuptial gift, reinforcing the interest in studying this farming insect as a new model for sexual selection.

## 1. Introduction

In species with internal fertilization, sperm storage in females—from sperm transfer to its use in fertilization—is a fundamental process of reproduction. Sperm storage includes three successive steps, involving both the male and the female [1]: (i) sperm transfer from the male reproductive tract to the receptive compartment of the female; (ii) migration of sperm to the site of storage for future fertilization; and (iii) use of only some spermatozoa for fertilization. In each of these stages, post-copulatory sexual selection occurs through sperm competition [2] and cryptic female choice [3,4]. As a result, the spatiotemporal dynamic of sperm storage is of great importance in understanding sexual selection [5–7], especially in arthropod females which have sperm storage organs [8]. In this regard, the study of both temporal and spatial [5,6] dynamics of sperm storage allows us to highlight processes through which post-copulatory sexual selection occurs.

Sperm precedence is one of these selective processes by which a male's sperm is favoured for fertilization, depending on its relative order of arrival in the female’s reproductive organs [9]. First male sperm precedence can be owing to the transfer of ‘mating plugs’ formed by either spermatozoa [10], coagulation of seminal fluids or male genitalia [11–13]. By contrast, last male sperm precedence can result from mechanisms of sperm displacement [5] or sperm stratification [14], favouring the last copulating male. In this process, the female can play a major role via sperm dumping/ejection which is a way to favour paternity of some males over others—i.e. to perform a cryptic female choice [4,15].

After mating and storing spermatozoa, the female will use them to fertilize oocytes. Consequently, there is a decrease in the amount of stored spermatozoa, which can lead to sperm limitation in some species—i.e. spermatozoa being a limited resource for the female [16]. This constraint can come from both quantity and quality, as spermatozoa may degrade over time during storage [17]. Sperm limitation not only affects egg production [18] but is also intricately tied to sexual selection as responses to sperm limitation may include selection for males producing/transferring more sperm, as well as females exhibiting multiple mating behaviour [19–21].

**Figure 1.**
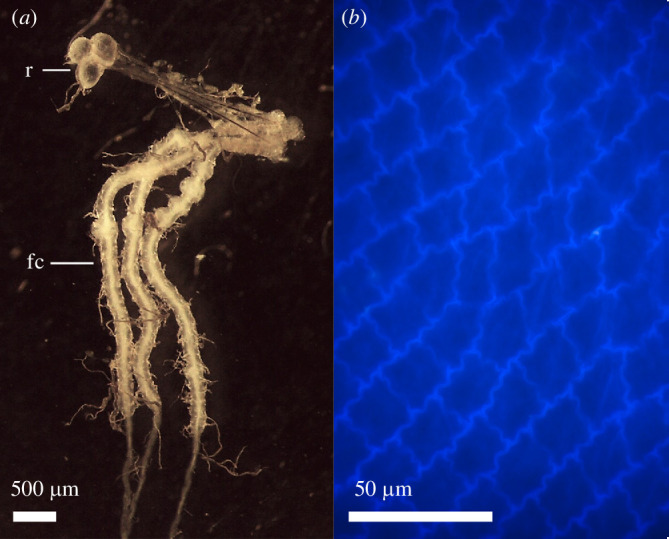
(*a*) The three spermathecae of a virgin female BSF and (*b*) the fishnet canal matrix under a fluorescence microscope. The colour of the matrix in (*b*) is owing to autofluorescence under UV light. Abbreviations: r, reservoirs; fc fishnet canals.

Among insects, the black soldier fly (BSF), *Hermetia illucens* (Diptera; Stratiomyidae), represents a source of economic interest [22,23]. BSF larvae are well known for their bioconversion capability, offering the possibility to reduce organic waste by transforming it to high-quality protein that can be used as feed for livestock and aquaculture [24,25]. However, the understanding of the biology of this species, particularly in its adult stage, remains limited [26]. It has been shown that BSF exhibits a polygynandrous mating system [27] and can lay multiple egg clutches [27]. Additionally, males respond to the risk of sperm competition by adjusting their production and allocation of spermatozoa depending on social contexts [28]. During their immobile mating, adults are attached for 20 min to over 1 h [29]. Moreover, BSF shows an interesting reproductive tract [30]. In females, each of the thin bases of the three separated spermathecae enlarges in a longer canal with a musculated outer wall and a fishnet-ornamented inner matrix (figure 1*a,b*; [30]). Then, a thinner canal embedded with muscles makes an elbow with a second rigid rod pierced with small holes preceding the sperm reservoir. Such an intriguingly complex structure raises questions about the reproductive processes of BSF. Recently, it has been demonstrated that mating in BSF enhances female longevity [31], implying the possible transfer of nuptial gifts. However, it is noteworthy that BSF does not transfer spermatophores during mating [30]. Therefore, if males do indeed provide a nuptial gift, the whole ejaculate—spermatozoa and seminal fluids—has to be investigated. This gift may be an endogenous genital gift [32], possibly including non-nutritional products similar to those found in *Drosophila melanogaster* seminal fluids [33].

Using a laboratory population of BSF, the present study aimed to investigate: (i) the spatiotemporal dynamics of sperm storage—from transfer to use; (ii) the variations in sperm quality along its storage in females; (iii) the ultrastructure of the spermathecae in relation to the mating status of the female; and (iv) the hypothesis of a nuptial gift transfer during mating.

## 2. Material and methods

### 2.1. Rearing conditions, mating and size measures

BSFs were reared and isolated as described in [30]. As BSF will not initiate mating when a single pair is placed in a cage (FM, personal observations, 2023), the first step to get matings consisted of transferring 20 virgin males to 15 × 15 × 15 cm cages containing 20 virgin females—considered as one series. Individuals remained in contact for 3 h to obtain mating pairs. Fifteen mating series were conducted for sperm storage dynamics description, six for the relationship between male size and fishnet canals volume, 13 for clutches monitoring, six for viability assessments and one for transmission electron microscopy (TEM).

BSFs were photographed under a Nikon SMZ745T stereomicroscope (x3.35 magnification) (Nikon, Japan) with a Leica IC 80 HD camera (Leica, Germany), their head width was measured using ImageJ. Head width is considered as a reliable proxy of the size of the individuals [34].

### 2.2. Sperm storage dynamics

To report the dynamics of sperm storage before females began laying eggs, flies were dissected (*n* = 128) at different intervals during and after mating. Preliminary observations showed that mating lasted 33.32 ± 10.19 min (mean ± s.e., *n* = 166) in the present experimental rearing. Considering this mating duration, females were dissected at 5 (*n* = 5), 10 (*n* = 15), 15 (*n* = 15), 20 (*n* = 15), 25 (*n* = 14) and 30 (*n* = 15) min after the beginning of mating and just after the end of mating (*n* = 12). Other females were dissected 24 h (*n* = 15) and 48 h (*n* = 10) after mating, before oviposition and after their first egg-laying (*n* = 12). Petri dishes in which females were isolated were examined with 4′,6-diamidino-2-phenylindole (DAPI) under a fluorescence microscope to investigate potential dumped sperm. Some females (*n* = 8) were also kept in ‘cages’ made of microscope slides (as in [35]) to reveal any dumped sperm.

Another set of females (*n* = 47) was dissected at the end of uninterrupted mating to describe the relationship between male size and the volume of the spermathecae just after mating.

To describe the spatial distribution of spermatozoa, the two compartments of the spermathecae were considered: the fishnet canals and the reservoirs (figure 1*a*). The fishnet canals were divided into two sections for spermatozoa counting: the first half and the second half. This allowed us to count spermatozoa in three distinct areas: the two halves of the fishnet canals and the reservoirs (figure 1*a*).

For all females, the abdomen was opened to collect the three spermathecae on a microscope slide in a drop of phosphate buffer saline. After dissections, spermathecae were photographed to take measurements of the fishnet canals’ width and length with ImageJ in order to estimate their volume using the formula of a cylinder: *V = πr*²*h*. The first measurements showed that volumes of the three fishnet canals were highly similar within a female (using the coefficient of variation (CV) which is calculated as CV = s.d./mean × 100, mean CV = 8.67 ± 5.20, *n* = 13) so finally only one fishnet canal was measured.

After taking a picture, a drop of DAPI (2 µg ml^−1^) was applied and incubated for 5 min without washing, then the spermathecae were crushed with a microscope coverslip to release spermatozoa and DAPI-labelled nuclei of spermatozoa were counted under a fluorescence microscope (Olympus CX40, Japan) with a ×20 objective. Spermatozoa counts were realized by visual analysis as in [28,30] and were repeatable (Pearson correlation coefficients: *r* = 0.91, *p* < 0.001, *n* = 12 samples counted two times). For some females that were dissected just at the end of uninterrupted mating (*n* = 13), spermatozoa were counted in the three spermathecae to ensure the number of spermatozoa transferred to each spermathecae was not variable (see results). Then, the number of spermatozoa was counted in one spermatheca and multiplied by three to estimate the total number of spermatozoa transferred. Sperm stored two days after mating and after the first egg-laying were counted in the three spermathecae.

### 2.3. Clutch monitoring

To evaluate how many eggs a female could lay, mating pairs (*n* = 114) were gently placed on the lid of a Petri dish and taken out of the cage until the end of mating. Time was recorded at the beginning and end of each mating to capture its total duration, and pairs were kept together at 24°C with cotton saturated with water in Petri dishes within which extra mating is not possible [30]. No food was provided to ensure that all females were in the same conditions for egg-laying and to prevent mould.

Pairs were observed every day to check for eggs. To collect eggs, females and males were transferred to a new Petri dish with a new cotton soaked with water. The eggs were gently separated with forceps to be counted by visual analysis under a Nikon SMZ745T stereomicroscope (x3.35 magnification) (Nikon, Japan) and then put in an incubator to check the fertility of the clutches at 27°C which was assessed by the visualization of the embryo red eye-spots before eclosion (electronic supplementary material, figure S1). Fertility was assessed before hatching because larvae that just eclosed disperse very quickly, which results in a mixture of eggs that is difficult to detect as hatched or not hatched. Some eggs were damaged during the counting process. Although it probably exists, partial infertility within a brood has never been obviously observed. The clutches were considered entirely fertile or infertile. This process was repeated until the death of the female which was then dissected. When monitoring clutches, spermatozoa were not counted; the reservoirs were examined to determine if spermatozoa were still present and to estimate the order of magnitude of the number of spermatozoa remaining.

### 2.4. Sperm viability assessment

Sperm viability in the fishnet canals was measured at two stages to assess if it could decrease after mating, before reaching the reservoirs. The assessment was conducted directly after mating (*n* = 25) and one day after mating (*n* = 26). Similarly, to evaluate whether sperm quality could be a factor limiting egg-laying, sperm viability in female reservoirs was measured at three stages: two days (*n* = 18), seven days (*n* = 10) and 14 days (*n* = 9) after mating.

The viability of spermatozoa was assessed using a live/dead sperm viability kit (Invitrogen). After dissection of the female, spermathecae were opened with forceps on a glass slide to be dipped in 5 μl of a 1 : 20 dilution of 1 mM SYBR−14. After 10 min of incubation in the dark, 5 μl of propidium iodide was added to the preparation. Pictures of the three fishnet canals/reservoirs were taken under a fluorescence microscope to blind count green (live) and red (dead) sperm nuclei. Spermatozoa that were half-green and half-red were counted as dead (electronic supplementary material, figure S2).

### 2.5. Transmission electron microscopy

Spermathecae from females of different mating statuses were investigated for ultrastructure through TEM: a virgin female, a female that mated on the day of its dissection and a female that mated two days before dissection. For each of them, semi-thin sections of their spermathecae—fishnet canals and reservoirs—were taken to describe their structure.

Spermathecae samples were fixed in a mixture of 2% paraformaldehyde (Merck, Darmstadt, Germany), 2% glutaraldehyde (Agar Scientific, France) and 0.1 M sucrose in 0.1 M cacodylate buffer (pH 7.4) for 24 h, washed 3 × 30 min in 0.1 M of cacodylate buffer and post-fixed for 1.5 h with 2% osmium tetroxide (Electron Microscopy Science, USA) in 0.1 M cacodylate buffer. After washing in 0.1 M cacodylate buffer for 20 min and 2 × 20 min in distilled H_2_O, samples were dehydrated in a graded series of ethanol solutions (50% ethanol for 2 × 15 min; 70% ethanol for 2 × 15 min and 1 × 14 h; 90% ethanol for 2 × 20 min; and 100% ethanol for 3 × 20 min). Final dehydration was performed by 100% propylene oxide (PrOx, VWR Int., France) for 3 × 20 min. Then, samples were incubated in PrOx/EPON epoxy resin (Fluka, Switzerland) mixture in a 2 : 1 ratio for 2 h with closed caps, in PrOx/EPON epoxy resin (Fluka, Switzerland) mixture in a 1 : 2 ratio for 2 h with closed caps and 1.5 h with open caps and in 100% EPON for 16 h at room temperature. Samples were replaced in new 100% EPON and incubated at 37°C for 24 h and at 60°C for 48 h for polymerization.

Semi-thin sections (thickness 0.8 µm) were cut with a ‘Leica Ultracut UCT’ ultramicrotome (Leica Microsystems GmbH, Wien, Austria), placed on glass slides, stained with Toluidine blue (Electron Microscopy Science, Hatfield, PA, USA) and embedded in Epon resin (Fluka, Switzerland) which was allowed to polymerize for 48 h at 60°C. The sections were then observed with a Nikon Eclipse 80i microscope (Nikon, Japan) connected to a DS-Vi1 camera driven by Nis-Element D 4.4 imaging software (Nikon, Japan).

Ultra-thin sections (thickness 70 nm) were placed on TEM one-slot grids (Agar Scientific, France) coated with Formvar film and stained for 20 min with 5% uranyl acetate (Electron Microscopy Science, Hatfield, PA, USA) and 5 min Reynolds lead citrate. The sections were then observed at 100 kV with a Jeol 1011 TEM (Tokyo, Japan) connected to a Gatan digital camera driven by Digital Micrograph software (GMS 3, Gatan, Pleasanton, CA, USA).

### 2.6. Statistical analyses

Wilcoxon–Mann–Whitney tests were used to compare the volume of fishnet canals and the number of spermatozoa counted during mating, as the data were not parametric. A Benjamini–Hochberg correction was applied to adjust *p*-values [36,37] owing to multiple comparisons across different dissection timings.

To assess variability in the number of laid eggs, a linear regression was performed with the log-transformed total number of eggs laid as the dependent variable. Covariates included male and female head width, the number of clutches of the female and mating duration.

Another linear regression was performed to analyse the mean volume of the fishnet canals just after mating. Covariates in this model included male and female head width, mating duration and the number of spermatozoa transferred.

A third linear regression was carried out to study female longevity. Covariates in this model were male and female head width, male longevity and mating duration.

A Wilcoxon–Mann–Whitney test was used to compare sperm viability percentages in the fishnet canals directly after mating and after one day. A Kruskal–Wallis test was used to compare sperm viability percentages in the reservoirs among females dissected 2, 7 and 14 days after mating. A paired-sample *t*‐test was used to compare the number of eggs between first and second clutches.

All statistical analyses were performed using R version 4.0.2 (R Core Team, 2024 [38]). The significance level was set at alpha = 0.05 for all tests. Residual uniformity of the linear models was assessed using the performance package [39]. Quantitative data were presented as mean ± s.d. when they were normally distributed and Q25 (first quartile); median; and Q75 (third quartile) when they were not.

## 3. Results

### 3.1. Sperm storage dynamics

**Figure 2.**
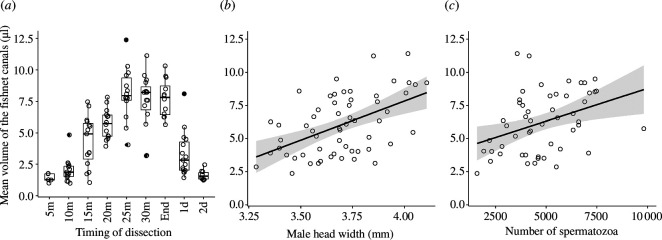
Mean fishnet canal volume in μl according to (*a*) the timing of dissection for interrupted matings, (*b*) male head width when measured at the end of uninterrupted mating, and (*c*) the number of spermatozoa in the fishnet canals at the end of uninterrupted mating. For (*a*), box plots show the median (horizontal bars), upper and lower quartiles (borders of the box). Whiskers extend from the 10th to the 90th percentiles. For (*b*) and (*c*) lines represent linear regression and shaded areas represent confidence intervals.

**Figure 3.**
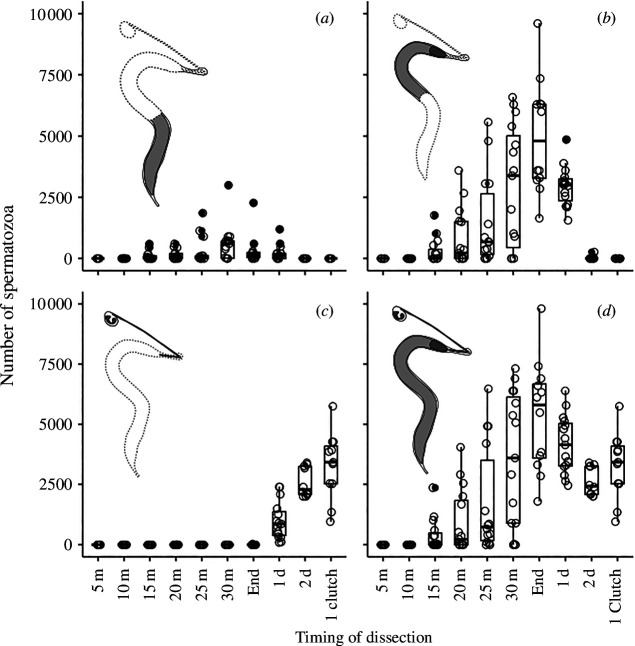
Number of spermatozoa according to the timing of dissection considering the three spermathecae (*a*) in the first half of the fishnet canals, (*b*) in the second half of the fishnet canals, (*c*) in the reservoirs of the spermathecae, and (*d*) in the totality of the spermathecae. Box plots show the median (horizontal bars), upper and lower quartiles (borders of the box). Whiskers extend from the 10th to the 90th percentiles.

At the early times of mating, 5 to 10 min after the beginning, the first part of the female spermathecae—the fishnet canals—was empty of seminal fluid (1.88 ± 0.86 μl; figure 2*a*) and almost empty of spermatozoa (Q25 = 0; median = 0; Q75 = 0 spermatozoa in the fishnet canals with *n* = 14 females having no spermatozoa and *n* = 1 female having eight spermatozoa (figure 3*a*)). After 15 min of mating, fishnet canals were swelled by seminal fluids transferred by the male (4.34 ± 2.02 μl; figure 2*a*) and reached their maximum volume between 25 min and the end of mating (7.79 ± 1.94 μl; figures 2*a* and 3). When taken out from the inside of the female tract, the handling of this seminal fluid with forceps made it look more like a jelly than a liquid fluid (electronic supplementary material, figure S4).

When measured at the end of uninterrupted mating, the mean volume of the fishnet canals was significantly and positively correlated to male head width (linear regression: *n* = 47, *F*_1,42_ = 26.70, *p* < 0.001, full model *R*² = 0.43, *β* ± s.e. = 6.03 ± 1.43; figure 2*b*) and the number of spermatozoa transferred (*F*_1,42_ = 6.67, *p* = 0.01, *β* ± s.e. = 4.04 ± 1.56; figure 2*c*). However, no significant relationship was found between the mean volume of the fishnet canals and female head width (*F*_1,42_ = 3.00, *p* = 0.09) or mating duration (*F*_1,42_ = 2.26, *p* = 0.14).

The number of spermatozoa transferred to each spermathecae (1659.5 ± 548.37 spermatozoa) was relatively similar across the three fishnet canals of a female (mean CV for the number of spermatozoa in spermathecae of a female = 15.36 ± 8.17) (electronic supplementary material, figure S5).

Spermatozoa were visible in the fishnet canals from some minutes upwards (at 15 min: 395 ± 635.18, Q25 = 0; median = 5; Q75 = 485 spermatozoa in the fishnet canals; figure 3*a,b,d*) and their number increased until the end of mating to reach a mean of 5346 ± 2290 (figure 3*d*). Most of the spermatozoa accumulated at the top of the fishnet canals (figure 3*c,d*)—336.2 ± 635.2, Q25 = 0; median = 135; Q75 = 255 spermatozoa in the first half of the fishnet canals and 5007.5 ± 2309.857 spermatozoa in the second half of the fishnet canals at the end of mating (Wilcoxon test: *p* < 0.001)—and no spermatozoa were observed in the reservoirs until the day after mating (figure 3*c*).

The total number of spermatozoa began to decrease 24 h after mating (4168 ± 1198.2 spermatozoa considering the entire spermathecae) and became significantly lower (Wilcoxon test: *p* < 0.01) 48 h after the end of mating even though females had not laid eggs (2634 ± 601.7, Q25 = 2100; median = 2430; Q75 = 3262 spermatozoa, which corresponds to 49% of the transferred spermatozoa). However, no dumped sperm was found in the Petri dishes or in microscope slide cages containing females; 24 h after mating, the spatial distribution of spermatozoa changed and the reservoirs began storing spermatozoa (spermatozoa in the reservoirs at the end of mating: 2.50 ± 8.66, Q25 = 0; median = 0; Q75 = 0 and 24 h after mating: 1000 ± 783.4; Wilcoxon test: *p* < 0.001; figure 3). At the same time, fishnet canals became progressively empty from spermatozoa (spermatozoa in the second half of the fishnet canals at the end of mating: 5007.5 ± 2309.86 and 24 h later: 2948 ± 816.44, Wilcoxon test: *p* = 0.01; figure 3*b*) and seminal fluid (mean volume at the end of mating: 7.74 ± 1.49 μl and 24 h later: 3.33 ± 1.78 μl, Wilcoxon test: *p* < 0.001) (figure 2*a*).

After a first egg-laying, the number of spermatozoa was not significantly different between the three reservoirs (ANOVA: *F*_1, 34_ = 0.11, *p* = 0.78), but the CV of the number of spermatozoa across the reservoirs of a same female was high (mean CV for the number of spermatozoa in the reservoirs of a female = 33.01 ± 27.63). No significant loss or gain of spermatozoa has been observed after the first egg-laying with 2634 ± 601.67, Q25 = 2100; median = 2430; Q75 = 3262 spermatozoa 48 h after mating and 3041.2 ± 1578.7 spermatozoa after first egg-laying.

### 3.2. Clutch monitoring

The mean of the total number of eggs laid by females after mating was 866.4 ± 355.7. From 114 matings, 47 single, 62 double and two triple clutches were obtained and three females did not lay at all. The total number of eggs laid varied significantly and positively with female head width (linear regression: *n* = 108, *F*_1,103_ = 32.81, *p* < 0.001, full model *R*² = 0.47, *β* ± s.e. = 0.35 ± 0.18) and negatively with male head width (*F*_1,103_ = 9.83, *p* < 0.01, *β* ± s.e. = −0.49 ± 0.16). The mating duration was not linked to the total number of eggs laid (*F*_1,103_ = 0.18, *p* = 0.67).

Females laid their first clutches 2.88 ± 1.09 d after mating. They laid more eggs (paired student test: *t* = 5.35, *p* < 0.001) in their first clutch (569.6 ± 163.3) than they did in their second clutch (483.1 ± 156.5) (electronic supplementary material, figure S6). Only two females laid third clutches containing, respectively, 85 and 505 eggs. On average, first clutches were laid at an age of 6.30 ± 1.93 d and second clutches at an age of 12.36 ± 2.44 d. The third clutches were laid when the females were 12 and 11 d, which was three days after the second clutches for both of them. There was no significant difference in fertility between the first (89% of fertile clutches) and the second clutches (78% of fertile clutches) (*χ*^2^-test: $\chi_{1}^{2}$ = 1.49, *p* = 0.22). All the females still had spermatozoa in their reservoirs after laying.

The longevity of females was significantly and positively related to their size (linear regression: *n* = 75, *F*_1,70_ = 9.80, *p* < 0.01, full model *R*² = 0.17, *β* ± s.e. = 3.89 ± 1.21). However, it was not significantly related to the size of the male they mated with (*F*_1,70_ = 1.96, *p* = 0.16) nor to mating duration (*F*_1,70_ = 0.97, *p* = 0.33). There was a slight but significant (*F*_1,70_ = 6.80, *p* = 0.01) positive relationship between the longevity of females and the longevity of the males they mated with (*β* ± s.e. = 0.14 ± 0.05).

### 3.3. Viability of spermatozoa

**Figure 4.**
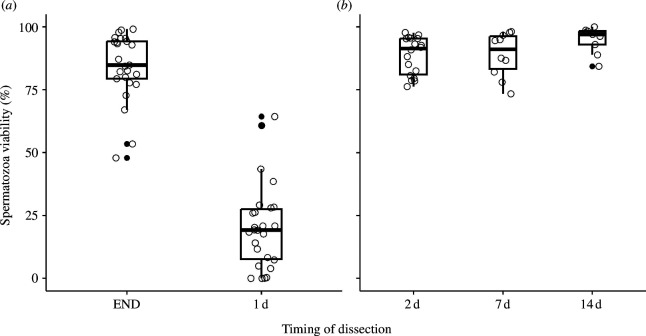
Percentage of viable spermatozoa counted (*a*) in the fishnet canals, and (*b*) in the reservoirs according to the timing of dissection. Box plots show the median (horizontal bars), upper and lower quartiles (borders of the box). Whiskers extend from the 10th to the 90th percentiles. Abbreviations: END*,* end of mating; 1 d, 1 day; 2 d*,* 2 days; 7 d*,* 7 days; 14 d*,* 14 days after mating.

In the fishnet canals, the proportion of viable spermatozoa was higher when counted directly at the end of mating compared to when counted one day after mating (Wilcoxon test: *W* = 646, *p* < 0.001), decreasing from 84 ± 13% to 20 ± 17% (figure 4*a*). In the reservoirs, the proportion of viable spermatozoa was high with a mean proportion of 90 ± 8% at the three time intervals. The proportion of viable spermatozoa in the reservoirs was not significantly different (Wilcoxon test: *p* = 0.796) when counted two days after mating (89 ± 7%) compared to when counted seven days after mating (89 ± 9%). In the same way, there was no significant difference (Wilcoxon test: *p* = 0.14) between counts realized seven days after matings and counts realized 14 days after mating (95 ± 5%). However, the proportion of viable spermatozoa counted 14 days after mating (95 ± 5%) was significantly higher (Wilcoxon test: *p* = 0.03) than the one counted two days after mating (89 ± 7%) (figure 4*b*).

### 3.4. Microscopy

**Figure 5.**
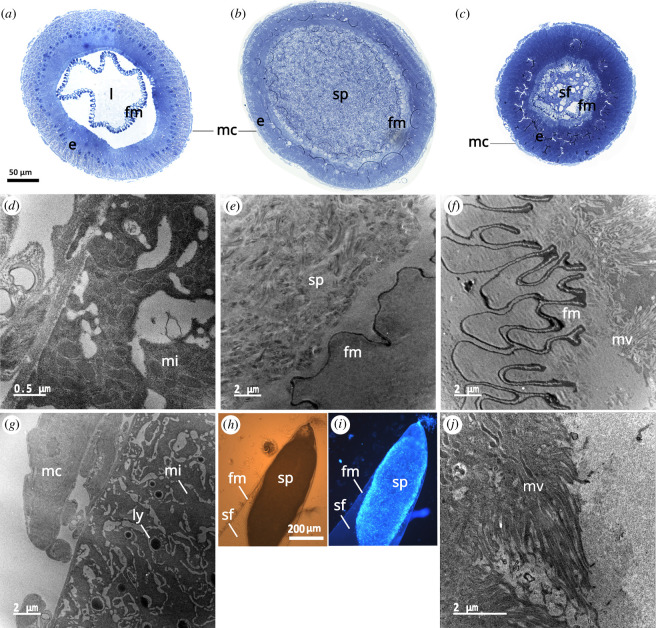
Semi-thin transversal sections of the fishnet canals (*a*) of a virgin female, (*b*) a female that just finished mating, and (*c*) a female that mated two days before. (*d*) and (*g*) outer part of the epithelium of a fishnet canal from a virgin female. (*e*) The inner part of a fishnet canal from a female that just finished mating. (*h*) and (*i*) light microscopy images of the sperm plug formed at the end of the fishnet canal with visible light and fluorescence—DAPI staining was used for (*i*) to show spermatozoa nuclei. (*f*) and (*j*) fishnet canal matrix and microvilli of the epithelium from a female that mated two days before. Abbreviations: fm, fishnet matrix; e, epithelium; l, lumen; mc, muscular cells; sp, spermatozoa; sf, seminal fluid; mi, mitochondria; mv, microvilli; ly, lysosomes-like structures.

The fishnet canals structure consisted of an epithelium that surrounded a matrix within which spermatozoa were transferred (figure 5*a*). The matrix was a thin sleeve floating in the lumen when empty (figure 5*a*), leaving a space between it and the epithelium. As seminal fluid was transferred, the matrix was swollen (figure 5*h,i*) to the point of being in contact with the epithelium (figure 5*b*), the inner layer of which is covered with microvilli (figure 5*f,j*). Two days after mating, it seemed that the matrix was still in contact with the microvilli (figure 5*c*). The outer layer of the epithelium was rich in mitochondria (figure 5*d,g*) and contained electron-dense structures that resembled lysosomes (figure 5*g*).

**Figure 6.**
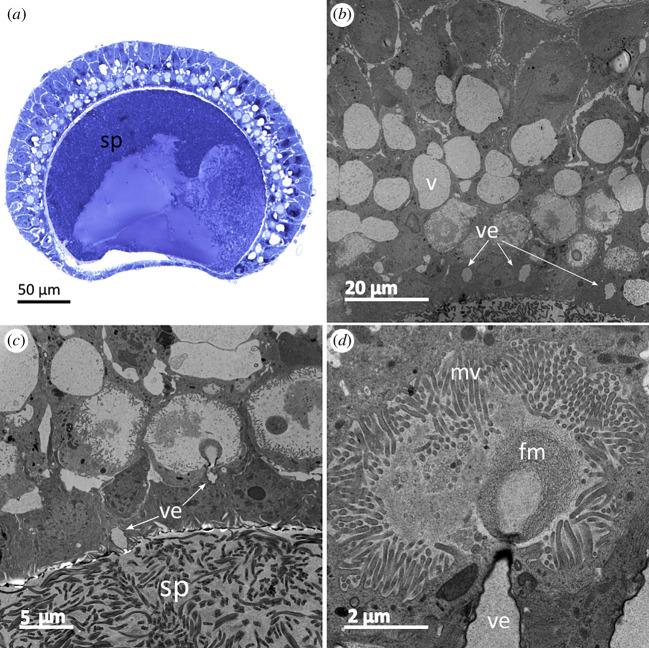
Ultrastructure of the reservoir of a female two days after mating. (*a*) Semi-thin section of the reservoir, (*b*) epithelium of the reservoir, (*c*) and (*d*) possible secretory compartment in the reservoir capsule. Abbreviations: sp, spermatozoa; v, vacuole; ve, vesicles; mv, microvilli; fm, fibrillar material.

Two days after mating, the fishnet canals were not fully empty as debris of seminal fluid could be seen (figure 5*c*). A proportion of the transferred spermatozoa were then in the reservoirs (figure 6*a,b*) where they were concentrated on one side of the capsule, leaving a space that may be filled with fluid. The wall cells of the reservoir were full of vacuoles (figure 6*a,c*) and cavities full of microvilli (figure 6*d*). These cavities contained an electron-dense fibrillar material connected to a vesicle (figure 6*c,d*). The area between the lumen containing the spermatozoa and the layer of cavities featured more of these vesicles, and some of them were connected to the lumen (figure 6*b,c*).

## 4. Discussion

In this study, the dynamics of sperm storage were monitored in female BSF, and the morphology and ultrastructure of the two main compartments of the spermathecae (the fishnet canals and the reservoirs) were analysed. A prolonged sperm storage process and the capacity of females to lay up to three fertile clutches were demonstrated. Additionally, it was found that sperm quantity and quality were not limiting factors for fertility. Interestingly, the morphology of the spermathecae suggests that sperm serves as a nutritive gift, digested by the female. These observations and their implications are discussed in detail here.

Sperm transfer in BSF appears to take place in two steps. The first one occurs at least within the first 15 min of mating and consists of the male transferring a seminal fluid devoid of spermatozoa which will swell the fishnet canals. The transfer of spermatozoa in the female’s spermathecae, more specifically in the fishnet canals, begins after 10 min of mating. In insects, seminal fluids are known to have considerable impacts on female physiology—among other traits, they affect oviposition rate, sexual receptivity and sperm storage [40–42]. Here, the seminal fluids looked more gelatinous in the spermathecae than they did when being ejaculated by the male (electronic supplementary material, figure S4). In other insects, some of these fluids play the role of mating plugs that retain the ejaculate within the female tract as they coagulate [11,12,43]. It seems that a similar process could take place in the spermathecae of BSF females. Whether this fluid could prevent sperm flushing or serve as a swimming support for spermatozoa is not known.

Interestingly, spermatozoa will accumulate and get stuck at the end of the fishnet canals during and after mating for 1 to 2 days before finally reaching the reservoirs of the spermathecae, contrary to what has been stated in [30]. Spermatozoa of BSF are long—3 mm [30], and their accumulation before access to the reservoirs resembles a mating plug made of spermatozoa—whose flagella make it look like a ball of wool (figure 5*h,i*). This apparent sperm plug may prevent the arrival of sperm from other males to the reservoirs in the context of multiple mating. It is particularly noteworthy that not all spermatozoa transferred by a male reach the reservoirs. Although the mechanism operating here is yet to be determined, this restricted access to the reservoirs strongly suggests that the junction between the fishnet canals and the reservoirs is a suitable site for female control over the amount of spermatozoa that goes into the reservoirs.

Because, in total, spermathecae contained more spermatozoa at the end of mating than two days after, it would seem that they are partly emptied between mating and oocyte fertilization, with possible sperm dumping at work here. The fishnet canals are completely emptied from spermatozoa and partially from seminal fluid 1 to 2 days after mating. However, while ejected sperm can be found in species that are known to do sperm dumping [5,35], such ejected sperm has not been observed in these experiments—either when searching in Petri dishes where females were stored or in ‘cages’ made of microscope slides (as in [35]).

Interestingly, TEM observations showed that the first part of the spermathecae, the fishnet canals, are structured like an insect gut. The fishnet canals are composed of muscular cells surrounding an epithelium and a matrix looking like a digestive peritrophic matrix [44,45]. The epithelium showed electron-dense structures that could be lysosomes as well as microvilli—structures that are characteristics of secretory and absorptive cells—surrounding the matrix full of sperm. Alongside the decreasing sperm number in the fishnet canals, sperm viability tests showed that the percentage of viable spermatozoa remaining in the fishnet canals decreased drastically the day after mating. Such a phenomenon has already been demonstrated in *Aedes aegypti* where it has been suggested to be the result of sperm digestion by the female [46]. The degradation of sperm observed in the fishnet canals may stem from a natural ageing process of sperm in a suboptimal environment. However, taken together—the structures observed through TEM, the disappearance of sperm from the fishnet canals that have not been found to be dumped and the altered viability of spermatozoa in the fishnet canals the day after mating—these results strongly indicate a phenomenon of sperm digestion. Given the extended lifespan observed in female BSF following mating, as documented by [31], it is conceivable for spermatozoa, seminal fluid or both to serve as nuptial gifts in BSF.

After mating, spermatozoa stored in the reservoirs were not a limiting resource for females’ fertility. Indeed, 54% of the females laid at least two clutches and less than 2% of the females (two females) laid three fertile clutches. All the females still had enough spermatozoa to lay at least one more clutch, and the mean number of spermatozoa in the reservoirs after one clutch would have been enough to lay approximately four more clutches. Moreover, sperm viability in the reservoirs remained constant over time. TEM observations of the reservoirs’ epithelium showed cavities covered with microvilli that may actively transport secretory substances towards the lumen (figure 6; [47]). We can hypothesize a possible female control over the reservoir environment to maintain spermatozoa in good condition [48,49]. Polyandry has been showed in this species [27,50], and even though the possibility of more clutches being laid in natural conditions cannot be ruled out, sperm limitation—either by their number or their viability—does not seem to be an explanation for these multiple matings. It can be hypothesized that, if a female’s capacity to lay clutches is not constrained by the quantity or the quality of stored sperm, the potential nuptial gifts transferred by males could serve as a motivation for multiple mating [51,52].

While larger females have been found to lay more eggs—which is common in many species, including insects [53], a counterintuitive observation is the negative relationship between male size and the number of laid eggs, even though male size is correlated with the amount of transferred seminal fluid which is known to enhance fertility in some species [54,55]. Interestingly, despite the positive correlation between male and female longevity, the lack of a significant relationship between male size and female longevity complicates the direct establishment of a link between the quantity of seminal fluid transferred by the male and its nutritional value.

Here, in addition to presenting the potential use of a sperm plug in BSF, a body of clues suggests that the male’s sperm could serve as an endogenous genital nutritive gift digested by the female directly in her spermathecae. The process described here represents a breeding ground for sexual conflicts [56], as strategies that are or may be used by both sexes—sperm plug in males and sperm digestion in females—may not maximize the fitness of their partner. Interestingly, if nuptial gifts are common in insects [32], endogenous genital ‘nutritive’ gifts [57] that could be used by the female to sustain her metabolic activities are less documented [57]. These findings reinforce the interest of BSF in the study of sexual selection.

## Data Availability

Data, script and supplementary materials used in this manuscript are provided by Frédéric Manas [58]. Supplementary materials are also available on Figshare: https://doi.org/10.6084/m9.figshare.27232461.v1. Data is also available as the electronic supplementary material [59].
